# Complete Genome Sequence of Pseudomonas plecoglossicida XSDHY-P, a Strain That Is Pathogenic for the Marine Fish *Larimichthys crocea*

**DOI:** 10.1128/MRA.01228-18

**Published:** 2018-10-04

**Authors:** Zhen Tao, Guoliang Wang, Suming Zhou

**Affiliations:** a School of Fisheries, Zhejiang Ocean University, Zhoushan, China; b School of Marine Sciences, Ningbo University, Ningbo, China; Loyola University Chicago

## Abstract

Pseudomonas plecoglossicida is a Gram-negative fish pathogen responsible for visceral granular disease in large yellow croaker, *Larimichthys crocea*. Here, we report the complete genome sequence of a verified virulent strain, XSDHY-P, that was isolated from spleen tissue of a diseased large yellow croaker.

## ANNOUNCEMENT

Pseudomonas plecoglossicida, a Gram-negative fish pathogen, was first isolated from a freshwater fish species, ayu (Plecoglossus altivelis) (1). The bacterium causes severe infections in ayu resulting in the syndrome of bacterial hemorrhagic ascites (BHA) (2, 3). More recently, P. plecoglossicida strains were also shown to be pathogenic to a maricultural fish species, large yellow croaker (LYC) (4). LYC infected by P. plecoglossicida often developed the clinical sign of white granulomas in liver, spleen, or kidney rather than BHA (4). The disease related to P. plecoglossicida was reported to cause a high mortality rate (70% to 80%) in LYC cultured in farms located in Eastern China (5). Currently, the disease still impacts LYC culture.

Studies are needed to not only elucidate the mechanisms used by P. plecoglossicida to invade and survive in the fish hosts but also facilitate a better understanding of the host immune responses so that new strategies can be developed to circumvent the spread of this pathogen to susceptible fish species. The P. plecoglossicida strain XSDHY-P was isolated from spleen tissue of a diseased LYC culture in Ningbo, China, and was verified to be particularly virulent toward LYC (4). To facilitate studies into P. plecoglossicida pathogenesis and the host response, the complete genome sequence of strain XSDHY-P was determined.

XSDHY-P was cultured in Luria-Bertani broth overnight at 30°C, and bacterial cells were collected by centrifugation. The genomic DNA (gDNA) was extracted with the QIAamp DNA minikit (Qiagen, Bethesda, MD, USA). The gDNA was further purified with a PowerClean DNA cleanup kit (Mo Bio Laboratories). The genome of strain XSDHY-P was sequenced using a PacBio RS II system (Pacific Biosciences, Menlo Park, CA) on single-molecule real-time (SMRT) cells using PacBio P6-C4 chemistry. The SMRT cells produced 929,227,527 bases generated through 130,030 subreads (*N*_50_ size, 13,697 bp; mean read length, 7,146 bp). All generated subreads were assembled in CANU version 1.5 with default parameters (correctedErrorRate, 0.045; corOutCoverage, 60) (6). The final assembly yielded a gapless completed circular chromosome sequence (168-fold coverage) with a G+C content of 62.66%, for a total of 5,525,520 bp. No plasmids were identified. A total of 5,103 genes, 4,806 protein-coding genes, 97 RNA-coding genes (22 rRNAs, 71 tRNAs, and 4 noncoding RNAs [ncRNAs]), and 200 pseudogenes were annotated using the NCBI Prokaryotic Genome Annotation Pipeline version 4.5 (7).

Analysis of the genome revealed that strain XSDHY-P contains genes for type III and type VI secretion systems, cell adhesion, and iron uptake. These features may facilitate its colonization and survival in fish hosts.

### Data availability.

The whole-genome sequence for P. plecoglossicida XSDHY-P has been deposited in DDBJ/ENA/GenBank under the accession number CP031146 (BioProject number PRJNA481851). The raw sequencing reads have also been submitted to the Sequence Read Archive (SRA) under accession number SRR7777440.
